# Novel compound heterozygous *EYS* variants may be associated with arRP in a large Chinese pedigree

**DOI:** 10.1042/BSR20193443

**Published:** 2020-06-02

**Authors:** Chunli Wei, Ting Xiao, Jingliang Cheng, Jiewen Fu, Qi Zhou, Lisha Yang, Hongbin Lv, Junjiang Fu

**Affiliations:** 1 Key Laboratory of Epigenetics and Oncology, The Research Center for Preclinical Medicine, Southwest Medical University, Luzhou, Sichuan, China; 2 State Key Laboratory of Quality Research in Chinese Medicine, Macau Institute For Applied Research in Medicine and Health, Macau University of Science and Technology, Taipa, Macau, Special Administrative Region of China; 3 Department of Ophthalmology, The Affiliated Hospital of Southwest Medical University, Luzhou, Sichuan, China

**Keywords:** Compound heterozygous variants, EYS gene, Missense mutation, Next generation sequencing (NGS), Retinitis pigmentosa (RP)

## Abstract

As a genetically heterogeneous ocular dystrophy, gene mutations with autosomal recessive retinitis pigmentosa (arRP) in patients have not been well described. We aimed to detect the disease-causing genes and variants in a Chinese arRP family. In the present study, a large Chinese pedigree consisting of 31 members including a proband and another two patients was recruited; clinical examinations were conducted; next-generation sequencing using a gene panel was used for identifying pathogenic genes, and Sanger sequencing was performed for verification of mutations. Novel compound heterozygous variants c.G2504A (p.C835Y) and c.G6557A (p.G2186E) for the *EYS* gene were identified, which co-segregated with the clinical RP phenotypes. Sequencing of 100 ethnically matched normal controls didn’t found these mutations in *EYS*. Therefore, our study identified pathogenic variants in *EYS* that may cause arRP in this Chinese family. This is the first study to reveal the novel mutation in the *EYS* gene (c.G2504A, p.C835Y), extending its mutation spectrum. Thus, the *EYS* c.G2504A (p.C835Y) and c.G6557A (p.G2186E) variants may be the disease-causing missense mutations for RP in this large arRP family. These findings should be helpful for molecular diagnosis, genetic counseling and clinical management of arRP disease.

## Introduction

It is well known that retinitis pigmentosa (RP) is a large group of genetically heterogeneous ocular dystrophies [1–4], including autosomal recessive (arRP), autosomal dominant (adRP), and X-linked inheritance (xlRP). The *EYS* (*Eyes shut homolog*) gene (GenBank access number: NM_001142800.1), also known as *SPAM, bA307F22.3, bA166P24.2, bA74E24.1, C6orf179, C6orf178, C6orf180, dJ1018A4.2, dJ22I17.2, EGFL10, dJ303F19.1, EGFL11*, or *RP25*, is mapped on human chromosome 6q12; the EYS protein (GenBank access number: NP_001136272.1) encodes is 3144-amino acids with a predicted molecular mass of 350 kDa. The EYS protein contains multiple epidermal growth factor (EGF)-like and LamG domains [5]. EGF-like domains have calcium-binding capability for protein–protein interactions, whereas LamG domains have binding sites for steroids, integrins, sulfatides, etc., which is important for maintaining the integrity of photoreceptor cells [6]. Indeed, the Eys knockout in zebrafish caused mislocalization of outer segment proteins, such as rhodopsin, opn1lw, opn1sw1, GNB3 and PRPH2, and disrupted actin filaments in photoreceptors [7].

Mutations in *EYS* (OMIM 612424) leading to retinitis pigmentosa 25 (RP25) for arRP (OMIM 602772) were first reported by two groups in 2008 [5,6]. Abd El-Aziz et al. analyzed six candidate genes within RP25 of 6q12 and identified six different *EYS* gene mutations in five unrelated families from ten Spanish pedigrees with arRP, leading to premature stop codons [6]. In another group, Collin et al. revealed homozygosity for a nonsense variant (p.Y3156X) and a deletion of 1 bp by analyzing the *EYS* gene in 10 arRP probands [5]. Mutations occurring in the EYS C-terminus in RP patients presented with hyperautofluorescent rings on fundus fluorescent photographs, elucidating genotype-phenotype correlations in EYS-associated RP (EYS-RP) [8]. More variants of the *EYS* gene were revealed [9–14] that caused changes in retinal structure and function [12,15,16].

Although the *EYS* gene mutations accounted for ∼5% of arRP in a cohort of RP patients who were mainly of western Europe ancestry [17], the *EYS* mutations of patients with arRP and genotype–phenotype correlations in the Chinese population have not been well described. Here, next-generation sequencing (NGS) was conducted to identify novel, compound heterozygous variants in *EYS* from a large Chinese arRP family, thereby extending its mutation spectrum.

## Materials and methods

### Pedigree and clinical assessment

**Figure 1:**
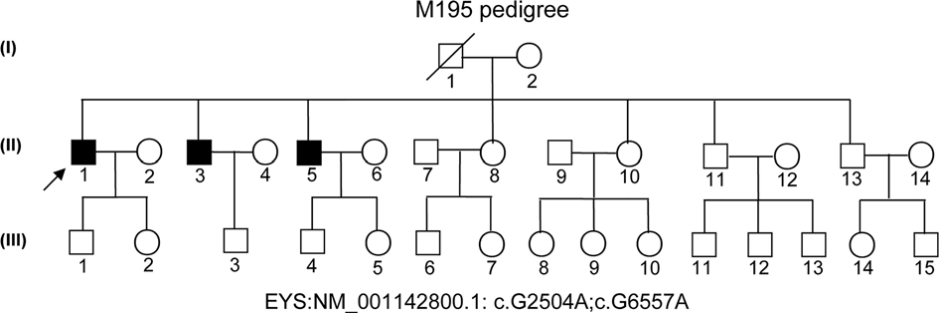
M195 pedigree with arRP Normal individuals are shown as empty circles (females) or squares (males), and affected individuals are shown as filled symbols. The patient designated by the arrow is the proband (II:1), who has the compound heterozygous variants of the *EYS* gene: NM_001142800.1: c.G2504A:p.C835Y; c.G6557A:p.G2186E.

This research study was carried out in accordance with the World Medical Association Declaration of Helsinki. This was approved by the ethical committee at the Southwest Medical University and written informed consent was obtained from all subjects. A Chinese proband (Figure 1, pedigree II: 1, arrow) was recruited. For clinical assessment, an ophthalmic examination was conducted, including best-corrected Snellen visual acuity, Humphrey visual fields, optical coherence tomography (OCT), slit-lamp biomicroscopy, fundoscopy, fundus photograph (FP) and fundus fluorescein photograph (FFP), as in previous studies [18,19].

### Sample collection and gDNA extraction

Fresh peripheral blood samples (2 ml each) from seven people in this family were collected, and gDNA was extracted by using our phenol/chloroform method that has been previously described [20–22]. One hundred healthy and ethnically matched individuals were sampled for DNA extraction as controls. A NanoDrop spectrophotometer (NanoDrop 2000, Wilmington, DC, U.S.A.) was used to measure the extracted DNA quality.

### Capture panel design, sequencing and data analysis

To characterize the disease-causing genes in the M195 family, TES (targeted next-generation sequencing) analyses were performed on the proband (M195), according to the Illumina paired-end libraries as reported previously [23,24]. The capture Agilent probes were applied in the previous reports [23–27], using a retinal disease capture panel. Then, paired-end sequencing Illumine reads were applied to align to the human hg19 reference genome by BWA (version 0.6.1) and the UCSC database [28]. Variations of SNPs and INDELs were refined to look for the causative mutations in suspected genes. The pathogenicity in each variant was estimated using the following programs: Mutation Taster, PolyPhen-2, SIFT, and I-Mutant2.0. The ExAC and HGMD databases were used to search for novel mutations.

### Primer design and PCR amplification

Locus-specific primer pairs (EYS-M195-16, EYS-M195-32), through the Primer 3 program with online website http://primer3.ut.ee/ containing mutations in the *EYS* gene, were designed [29] (Table 1). PCR products for *EYS* that were 374 bp and 478 bp in length were amplified using gDNA as a template. PCR amplification and DNA sequencing in the *EYS* variants were used to all the available gDNA for variant verification and segregation analysis [19,30].

**Table 1:** The sequences of PCR primers and PCR product sizes

Primer name	Left primer	Sequence (5′-3′)	Right primer	Sequence (5′-3′)	Size	°C
EYS-M195-16	EYS-M195L16	tggatggactggacagaact	EYS-M195R16	gtcccctacccacaatgtaca	374	60
EYS-M195-32	EYS-M195L32	cagtcttttcctctgtactggt	EYS-M195R32	cttcatgcactggtctggaa	478	60

### Sanger sequencing and co-segregation analysis

All the PCR products were then directly used for Sanger method sequencing using a machine of ABI-3500DX sequencer from Applied Biosystems Inc. in our laboratory through the specific primers EYS-M195L16 or EYS-M195L32 (Table 1). All controls with unrelated ethnical-matched were also used to sequence by aforementioned primers in Table 1. For co-segregation, we conducted an analysis based on our sequenced results and the patient’s clinical phenotype.

### The structure of the EYS protein and bioinformatics

A search of conserved domains from coding nucleotide sequences or proteins by inputting ‘EYS’ was performed using an online NCBI system (Conserved Domain Search website: https://www.ncbi.nlm.nih.gov/Structure/cdd/wrpsb.cgi?INPUT_TYPE=precalc&SEQUENCE=224451128) [31,32]. Homologs were determined by the online NCBI system for the EYS protein: https://www.ncbi.nlm.nih.gov/homologene?Db=homologene&Cmd=Retrieve&list_uids=129971.

### RNA expression profiles

The *EYS* mRNA expression profiles in 27 normal human tissues were obtained by RNA-sequencing to determine tissue-specificity through an online NCBI database (https://www.ncbi.nlm.nih.gov/gene/346007#gene-expression) [33]. Since no retinal tissue was obtained from RNA sequencing, the *EYS* mRNA expression profiles in different tissues, including the human retina were also obtained from the three transcriptomic datasets (https://www.proteinatlas.org/ENSG00000188107-EYS/summary/rna).

## Results

### Pedigree and clinical characteristics

**Figure 2:**
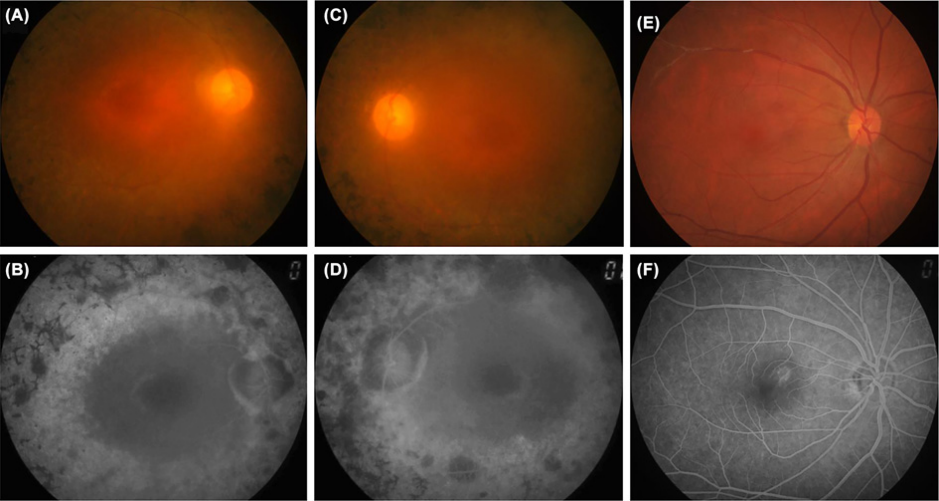
Representative fundus photographs (FP) and fundus fluorescein photographs (FFP) of the proband II:1 from both eyes (**A** and **B**) FP and FFP of the proband in the right eye respectively. (**C** and **D**) FP and FFP of the proband in the left eye respectively. (**E** and **F**) Normal control images for FP and FFP in the right eye, respectively.

A large Chinese pedigree, consisting of 31 members including a proband and two additional patients was recruited (Figure 1, pedigree II: 1 with arrow indicated). The proband, who was from a non-consanguineous family, is a 56-year-old male, who first showed night blindness symptom at 15 years of age. The FPs and FFPs of the proband (II:1) in both eyes and control images are shown in Figure 2. The proband clearly showed clear severe RPE atrophic changes, pigmentation with bone spicules in the retina of the peripheral-mid and transparent macula. The vessels were extremely small, and the optic disc was pale or waxen in both eyes (Figure 2A–D). Electroretinography (ERG) showed reduced cone and core responses or low amplitude ERG in the patient (data not shown). Two younger brothers of the proband showed similar RP feature. The proband’s parents and other family members didn’t exhibit any RP features, suggesting an autosomal recessive inheritance pattern. Based on pedigree analysis, the cases in this family are likely considered arRP.

### NGS analysis and screening of pathogenic mutations

Causative mutations were revealed by targeted capture high-throughput sequencing (Figure 1, pedigree II: 1) [23,24]. Compound heterozygous variants, including a missense variant c.G2504A in exon 16 and a missense variant c.G6557A in exon 32 of the *EYS* gene (NM_001142800.1, isoform 1) in the M195 proband, were identified, leading to amino acid substitutions, from Cysteine (Cys, C) to Tyrosine (Tyr, Y) at position 835 (p.C835Y), and from Glycine (Gly, G) to Glutamic acid (Glu, E) at position 2186 (p.G2186E) of the EYS protein (NP_001136272.1), respectively (Figure 1 II: 1). The possible deleterious and pathogenic variants: c.G2504A (p.C835Y) and c.G6557A (p.G2186E) in the *EYS* gene are presented in Table 2.

**Table 2:** Characteristics of *EYS* variants and analysis of disease-causing effects

Gene	Exon	Variation				Polyphen-2	Mutation Taster	I-Mutant2.0	SIFT	ExAC
		Nucleotide*	Protein*	Type	Status					
EYS	16	c.G2504A	p.C835Y	Missense	Heter	B(0.012)	DC (0.999)	DS	T(0.3)	Novel
	32	c.G6557A	p.G2186E	Missense	Heter	PD (0.98)	DC (0.999)	DS	T(0.31)	known

Abbreviations: B, benign; c, variation at cDNA level; DC, disease causing; DS, decreased stability; ExAC, Exome Aggregation Consortium; EYS, eyes shut homolog; Heter, heterozygote; p, variation at protein level; PD, probably damaging; T, tolerated.* All nucleotides and amino acids are abbreviated according to the International Union of Pure and Applied Chemistry (IUPAC).

### Variant confirmation and analysis of co-segregation results

**Figure 3:**
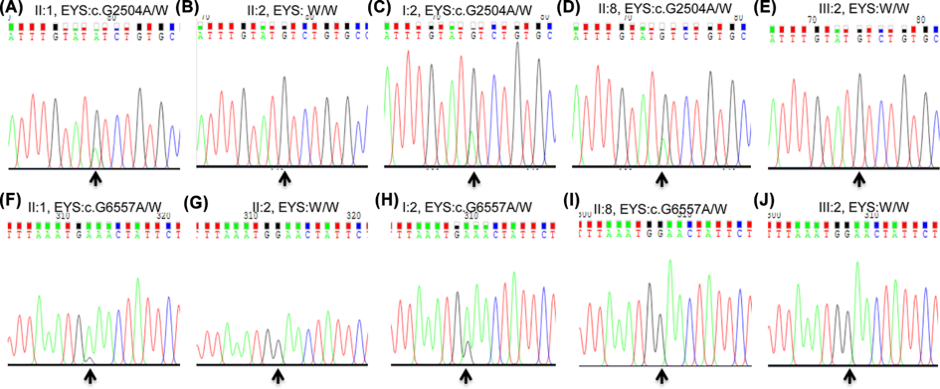
Photogram profiles for *EYS* verification by Sanger sequencing Panels (**A–E**) indicate the sequencing results for the *EYS* mutant allele of the c.G2504A or its wild type, whereas panels (**F–J**) indicate the sequencing results for the *EYS* mutant allele of the c.G6557A or its wild-type. The arrows indicate mutations at the nucleotide positions: c.G2504A or c.G6557A in the *EYS* gene. W, wild-type allele.

Although deficient, variant confirmation and analysis of co-segregation for *EYS* were conducted by Sanger sequencing (Figure 3). The c.G2504A and c.G6557A variants of *EYS* were confirmed in the mutant compound heterozygous types in the proband (pedigree II: 1; Figure 3A,F); and we revealed wild-type without RP in the proband’s wife (pedigree II: 2; Figure 3B,G), mutant heterozygous types without RP symptoms in the proband’s mother (pedigree II: 2; Figure 3C,H) and mutant heterozygous types without RP symptoms in his younger sister (pedigree II: 8; Figure 3D,I), wild-type without RP symptoms in the proband’s daughter (pedigree III: 2; Figure 3E,J), and mutant compound heterozygous types in the proband’s two younger brothers (II: 3 and II:5 with RP symptoms, data not shown). Thus, these findings show co-segregation with disease in this family we tested, and suggest its role in pathogenesis. Furthermore, 100 ethnically matched normal controls were sequenced for both variants of *EYS*; no variants were revealed (data not shown).

### Effects of function in EYS variants c.G2504A (p.C835Y) and c.G6557A (p.G2186E)

**Figure 4:**
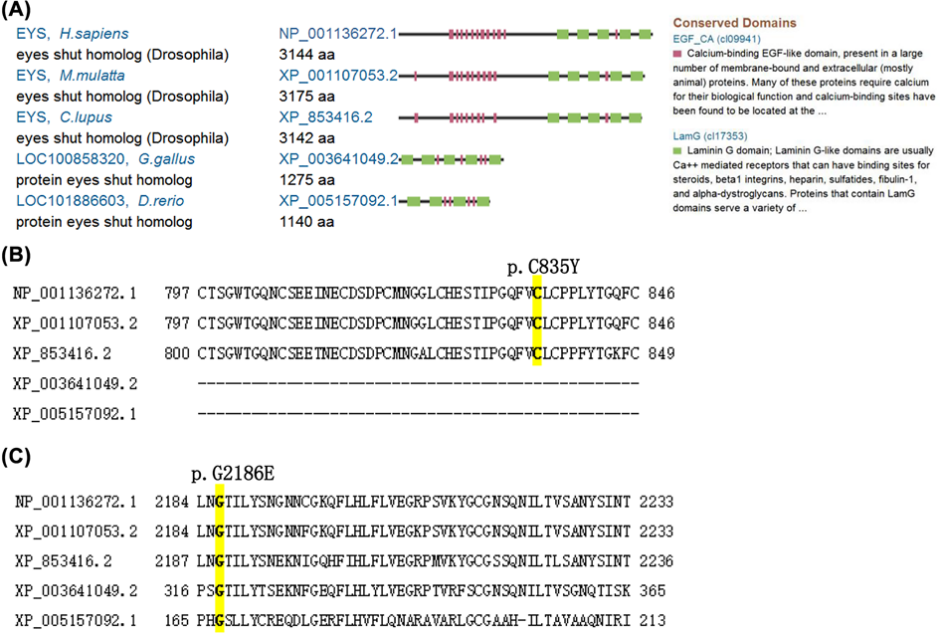
EYS structure and conservation domains (**A**) EYS ortholog, structure and domains. (**B**) Comparison of the conservation domain of EYS and the position of variant p.C835Y. (**C**) Comparison of the conservation domain of EYS and the position of variant p.G2186E. Conserved amino acids are highlighted in yellow.

Searching of the Conserved Domain Database (CDD) from NCBI was performed. Comparing human EYS protein to other species indicated that EYS is conserved among chicken, dog, rhesus monkey, and zebrafish (Figure 4A). EYS has two repeated conserved domains: calcium-binding EGF-like domain (EGF_CA, cl09941) and laminin G domain (LamG, cl17353). EYS variant p.C835Y, located in the EGF_CA domain (amino acid 810∼846), and p.G2186E, located in the lamG domain (amino acid 2149∼2315), were also highly conserved (Figure 4B,C), which may affect protein function. However, no *EYS* ortholog in mice was found.

MutationTaster revealed disease-causing, SIFT revealed tolerated, and I-Mutant2.0 showed decrease stability for both p.C835Y) and p.G2186E variants; Polyphen 2 for p.G2186E showed probably damaging, whereas Polyphen 2 for p.C835Y showed benign (Table 2). Thus, these compound and heterozygous variants c.G2504A (p.C835Y) and c.G6557A (p.G2186E) in EYS might damage protein function in this arRP family. The variant c.G2504A (p.C835Y) revealed a novel mutation even though c.G6557A (p.G2186E) was not found [16] in a search of in the HGMD and ExAC databases (Table 2).

Comprehensively, the present study shows that compound heterozygous, pathogenic missense variants of *EYS* c.G2504A (p.C835Y) and c.G6557A (p.G2186E) may cause arRP in this large Chinese pedigree.

### Expression profiles of *EYS* mRNA

**Figure 5:**
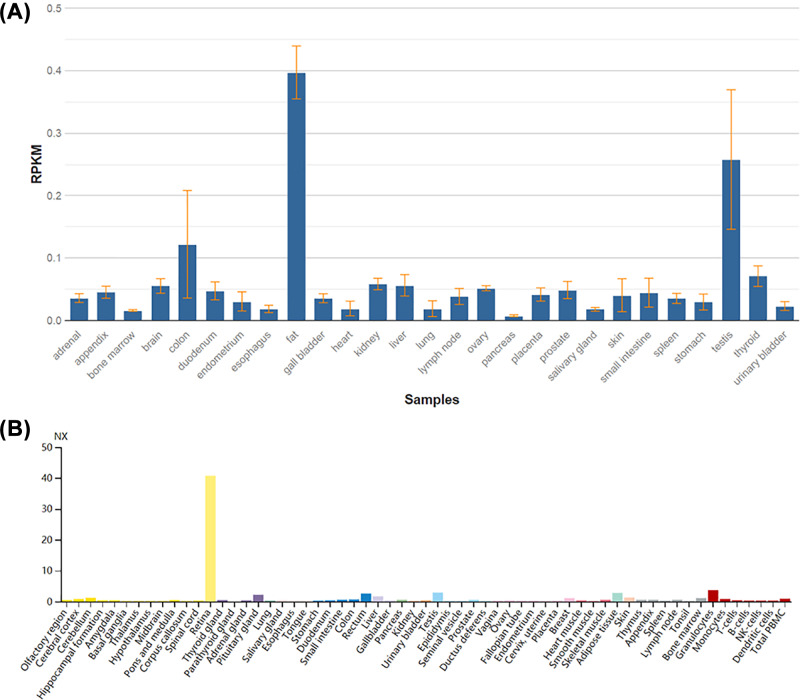
*EYS* mRNA expression profiles (**A**) Expression profiles for *EYS* mRNA in 27 indicated human samples. RPKM: Reads Per Kilobase Million. (**B**) Expression profiles for *EYS* mRNA in 55 indicated tissue types and 6 blood cell types; NX, consensus normalized expression.

RNA-seq data showed that *EYS* has low expression in 27 tested different human tissues but fat has highest expression (RPKM value: 0.397 ± 0.042), followed by the testis (RPKM value: 0.258 ± 0.112); the pancreas has the lowest expression (RPKM value: 0.008 ± 0.002) (Figure 5A and Table 3). The protein is expressed in the photoreceptor layer of the retina [34,35]. However, no eye tissue data were shown by RNA-seq; no mouse EYS ortholog existed. Then the *EYS* mRNA expression profiles in more different tissues, including the retina and cells (55 tissue types and 6 blood cell types) were also obtained, and the results showed that *EYS* is most highly expressed in the retina of humans, with an NX value of 40.7 in the retina, but an NX value of only 2.9 in the testis (Figure 5B). Thus, this demonstrated that EYS is only highly expressed in retinal tissue and probably only plays a vital role in the retina of the eye.

**Table 3:** Expression of *EYS* mRNA in human different tissues

Sample	Numbers	RPKM values	Counts
Adrenal	3	0.036 ± 0.007	5527
Appendix	3	0.045 ± 0.01	6330
Bone marrow	4	0.016 ± 0.001	6091
Brain	3	0.055 ± 0.012	9646
Colon	5	0.122 ± 0.086	61,808
Duodenum	2	0.047 ± 0.014	4398
Endometrium	3	0.031 ± 0.015	5475
Esophagus	3	0.019 ± 0.006	4820
Fat	3	0.397 ± 0.042	61,275
Gall bladder	3	0.036 ± 0.007	9284
Heart	4	0.019 ± 0.012	7054
Kidney	4	0.058 ± 0.009	11,252
Liver	3	0.056 ± 0.017	9539
Lung	5	0.019 ± 0.013	5828
Lymph node	5	0.038 ± 0.013	17,239
Ovary	2	0.052 ± 0.004	10,458
Pancreas	2	0.008 ± 0.002	1487
Placenta	4	0.042 ± 0.011	14,889
Prostate	4	0.049 ± 0.014	9969
Salivary gland	3	0.018 ± 0.003	5785
Skin	3	0.041 ± 0.026	11,209
Small intestine	4	0.045 ± 0.023	9755
Spleen	4	0.035 ± 0.008	10,262
Stomach	3	0.03 ± 0.013	6032
Testis	7	0.258 ± 0.112	1,79,148
Thyroid	4	0.071 ± 0.016	26,332
Urinary bladder	2	0.023 ± 0.007	3920

## Discussion

The EYS protein (NP_001136272.1) has different isoforms. Consistent with RNA expression data (Figure 5), expression analysis by PCR of cDNA from tissues and cell lines showed that the *EYS* gene is only expressed in the retina and in a retinoblastoma cell line in humans [6]. Immunohistochemistry (IHC) in mature pig retinas showed Eys expression at the photoreceptor layer. In a zebrafish model, Eys absence led to degeneration in the photoreceptor outer segments, photoreceptor death, disorganized retinal architecture, decreased ERG responses and caused visual dysfunction [7,36,37], whereas in humans, by novel EYS mutations, IHC revealed advanced retinal degenerative changes of in all eyes with rod photoreceptor absence [38]. Collin et al. in 2008 independently identified a large transcript, encoding 3165 amino acids (isoforms 4, NP_001278938) with a signal peptide, and domains with EGF-like and laminin A G-like. All four *EYS* transcripts were expressed in the retina and the Y79 cell line of humans, whereas isoforms 2 and 3 of *EYS* are also expressed in the testis [35]. BLAST analyses found that this *EYS* gene was the true ortholog of the Drosophila ‘*eyes shut’* (*eys*) gene. Surprisingly this *EYS* gene is abundantly expressed in the retina in humans, but *Eys* is completely absent in mouse. Expression profiles of *EYS* mRNA showed that it is only highly expressed in the retina tissue of humans but not in other tissues (Figure 5), demonstrating that EYS probably only plays a vital role in the retina of the eye.

The targeted NGS approach has been proven to be efficient in the genetic diagnosis of RP [9,10,30]. In this paper, we identified that compound heterozygous, missense variants (c.G2504A, p.C835Y; c.G6557A, p.G2186E) of *EYS* may cause arRP in the large Chinese family by targeted NGS. Analyses by MutationTaster revealed disease causing, SIFT revealed tolerated, and I-Mutant2.0 showed decrease stability for both variants; Polyphen 2 for p.G2186E was probably damaging, whereas Polyphen 2 for p.C835Y was benign. Thus, combined all information, these compound and heterozygous variants in *EYS* might be pathogenic in this large Chinese pedigree by damaging and reducing the stability of the EYS protein. Variant p.C835Y for EYS is located in a conserved EGF_CA domain that may be crucial for protein–protein interactions, whereas variant p.G2186E for EYS is located at a conserved LamG domain that has the role in signal transduction, adhesion, migration and differentiation by mediating cell adhesion molecule through binding. In this regard, the *EYS* variants p.C835Y and p.G2186E may interrupt protein–protein interactions and signal transduction, leading to the pathogenesis of arRP. To the best of our knowledge, the *EYS* mutation of c.G2504A (p.C835Y) is novel and enriches the *EYS* mutation spectrum, and is linked with arRP phenotypes. Therefore, these findings will help in understanding the molecular pathogenesis of RP for diagnosis, prevention as well as genetic counseling.

## Conclusion

In summary, our study identified the compound heterozygous variants c.G2504A (p.C835Y) and c.G6557A (p.G2186E) of EYS, which may cause arRP in this large Chinese family. It is the first study to reveal the novel *EYS* mutation (c.G2504A, p.C835Y) for RP disorder in our Chinese patients, expanding the *EYS* mutation spectrum. These findings should help in understanding the molecular pathogenesis of arRP disease for diagnosis, clinical management and genetic counseling.

## Abbreviations

arRP: autosomal recessive retinitis pigmentosa
adRP: autosomal dominant retinitis pigmentosa
EGF: epidermal growth factor
FP: fundus photograph
FFP: fundus fluorescein photograph
RP25: retinitis pigmentosa 25
xlRP: X-linked inheritance retinitis pigmentosa
